# Efficacy and tolerability of injectable sodium valproate in patients with mania

**DOI:** 10.4103/0019-5545.31603

**Published:** 2006

**Authors:** R. K. Solanki, Paramjeet Singh, Renu Khandelwal, Aarti Midha

**Affiliations:** * Professor and Unit Head-III Department of Psychiatry, SMS Medical College, Jaipur, Rajasthan; ** Assistant Professor Department of Psychiatry, SMS Medical College, Jaipur, Rajasthan; *** Resident Doctor – Final year Department of Psychiatry, SMS Medical College, Jaipur, Rajasthan; **** Resident Doctor – Final year Department of Psychiatry, SMS Medical College, Jaipur, Rajasthan

## Abstract

**Background:**

Sodium valproate is among the newer mood stabilizers and is also an anticonvulsant.

**Aim:**

To assess the effect of intravenous sodium valproate in patients with acute manic episodes of bipolar disorder.

**Methods:**

A 1-week open trial was conducted in the year 2004–2005 at the emergency ward of the Psychiatric Centre, SMS Medical College, Jaipur, in which 30 patients participated.

**Conclusion:**

Substantial improvement was seen. No major side-effects were noted except marginal elevation of the SGOT and SGPT. The findings suggest that injectable sodium valproate is a safe and effective mood stabilizer for patients with mania.

## INTRODUCTION

Acute manic excitement is disturbing for patients, their relatives and society. Rapid resolution of acute manic episodes can reduce the substantial personal and economic burden on patients, their relatives and society.

Treatment of bipolar disorder includes anticonvulsants and antipsychotics.^1^–^5^ Conventional neuroleptics such as haloperidol and chlorpromazine and novel antipsychotics such as risperidone, olanzapine and ziprasidon are effective in treating acute mania.^1^,^4^,^6^ The efficacy of neuroleptics in treating manic symptoms is related to their D_2_ receptor blockade, while the efficacy of mood stabilizers is related to their own specific mechanism such as blockage of synthesis of IP_3_ with lithium, and that of the GABAergic system with sodium valproate.^4^,^5^,^7^,^8^

In the USA, the anticonvulsant divalproex sodium is widely used because of its proven antimanic activity and evidence of long-term protective effect against recurrence of bipolar disorder. Its effects are dependent on the dose and serum concentration.^2^,^4^,^8^. To exert an antimanic effect, the plasma therapeutic level should be in the range of 45–150 mg/L.^9^ It has been claimed that rapid resolution of manic symptoms can be achieved with a loading dose of 20 mg/kg/day, which starts after 5 days of therapy.^10^ Due to gastrointestinal intolerance and poor compliance in some of the acutely ill patients it was not possible to administer an oral loading dose, hence the injectable route was used.^10^–^12^

This study proposed to test the efficacy of injectable sodium valproate as compared with oral sodium valproate.

## METHODS

The current study assessed the efficacy and safety of injectable sodium valproate in an open-label trial, parallel group, randomized comparative prospective study in subjects with acute manic excitement. Thirty patients completed the study. The subjects were recruited from the OPD, Psychiatric Centre, SMS Medical College, Jaipur on the basis of inclusion and exclusion criteria after taking written consent, both from the subjects and their relatives.

### Inclusion criteria

- Patients between the ages of 18 and 65 years
- Patients who fulfilled the ICD-10 criteria for bipolar affective disorder
- A score of at least 20 on the 11-item Young Mania Rating Scale (YMRS) at screening^13^
- Patients who had not taken any treatment since the past 2 months
- Patients who provided written informed consent

### Exclusion criteria

- Patients with a YMRS score of less than 20
- Patients with an organic brain syndrome
- Patients with schizophrenia, schizoaffective disorder, epilepsy or any other neurological disorder
- Patients with a history of alcohol and drug abuse within 3 months from the start of the study
- Patients at imminent risk of causing injury to themselves or others
- Patients with serious or unstable medical illnesses
- Patients with marked laboratory abnormalities especially liver and renal function tests and haemogram
- Patients with a history of severe drug allergy or hyper-sensitivity
- Pregnant and nursing women and those with childbearing potential not on adequate contraception
- Patients with a significant history of previous liver and renal diseases
- Recently relapsed cases were excluded on account of having uniformity of cases in the study group.

### Baseline investigations

- Complete physical examination
- Complete haemogram; pregnancy test
- Liver function tests
- Renal function tests
- ECG

Assessments were done at screening, baseline, day 3 and day 7 by using the YMRS^13^ and clinical global impression scale.^15^

Selected patients were admitted in the emergency ward after complete physical examination and baseline investiga-tions. Each case was recorded in detail on a specially designed proforma, which included the consent form, identification data, sociodemographic data and detailed history of bipolar illness and baseline investigations. Patients in the experimental group were given single-blind treatment with either injectable sodium valproate + oral risperidone while those in the control group received oral sodium valproate + oral risperidone.

In the exclusion criteria, a YMRS score less than 20 was an arbitrary dividing line indicating less severe psychopathology, so subjects who scored more than 20 were randomly assigned to two groups.

Injectable sodium valproate was given in dose of 1500–2000 mg/day in divided doses by IV infusion with 500 cc of 5% GDW (maximum 1000 mg at a time).

The dose of oral sodium valproate was also in the range of 1500–2000 mg/day in divided doses. Risperidone can be given in a dose of 4–8 mg/day according to the patient's requirement.

The study medication is available as a parenteral preparation of 5 ml/vial containing 100 mg sodium valproate per ml. The study medication was given in as flexible a schedule as permissible. The investigator had the option to adjust the daily dose depending on individual patient's response or presence of intolerable adverse effects. Pretreatment signs and symptoms, concomitant medications and adverse effects were recorded. Serious adverse events, if any, were recorded separately; vital signs, including the blood pressure, pulse, body weight were recorded daily. Laboratory parameters such as the haemogram, SGOT, SGPT, serum bilirubin, serum urea and serum creatinine were also recorded at baseline, day 3 and day 7.^11^,^12^,^16^,^17^

The serum sodium valproate levels were measured on day 3 and day 7.

### Concomitant medications

1. No other psychotropic medication was allowed during the trial.
2. Drug treatment for somatic illnesses was limited as much as possible.
3. Injectable/oral lorazepam was used as and when required in a dose of up to 2–4 mg/per day.

## RESULTS

The results of the paired t test demonstrate a highly significant reduction in the YMRS scores of patients on injectable sodium valproate compared with those on oral sodium valproate as shown in Tables 1–3.

**Table 1:** YMRS score of experimental and control groups

Group	*n*	Mean YMRS at the time of admission	Mean YMRS on day 3	Mean YMRS on day 7
Experimental	30	38.0848	20.0178	15.8441
Control	30	44.0789	31.2458	21.1804

**Table 2:** Significance of difference on YMRS score on day 3

Group	*n*	Mean YMRS on day 3	Standard deviation	Standard error of mean	*t*	df	P
Experimental	30	20.0178	3.32	0.24	-9.979	49	<0.01
Control	30	31.2458	2.98	0.25			

**Table 3:** Significance of difference on YMRS score on day 7

Group	*n*	Mean YMRS on day 7	Standard deviation	Standard error of mean	*t*	df	P
Experimental	30	15.8441	4.37	0.311	4.765	49	<0.01
Control	30	21.1804	4.61	1.015			

In the experimental group, 18 patients showed more than 50% decrease in YMRS scores, 6 patients showed minimal improvement (less than 25%), 4 patients showed no changes and 2 patients become worse at the end of the trial (because of psychotic features).

In the control group, 14 patients showed more than 50% decrease in YMRS scores and 4 patients did not show any changes. In 7 patients the decrease in YMRS score was less than 25%.

### Safety

No serious adverse effects were recorded in any patient in the experimental group. In general, adverse effects were mild in intensity and included marginal (benign) elevation of liver enzymes (SGOT and SGPT) to less than 140 IU per ml in 24 patients, flatulence in 1 patient, diarrhoea in 2 patients, vomiting in 3 patients, nausea in 5 patients, thrombophlebitis in 1 patient. Oral loading of sodium valproate resulted in significant elevation of serum SGOT and SGPT (more than 140 IU per ml).

No clinically relevant changes in pulse, blood pressure and heart rate were seen throughout the trial. In addition, there were no clinically relevant abnormalities of the bio-chemical and haematological parameters.

## DISCUSSION

Only injectable antipsychotics and benzodiazepines are used to treat acute manic excitement; these have their own limitations. Oral antiepileptics, which are also used, also have their limitations such as poor treatment compliance and GI intolerance. As these patients pose a special problem of compliance in hospital, several studies have recommended an oral loading dose of valproate for such patients, which again has limitations of compliance and GI intolerance.^10^–^12^ The authors used injectable valproate to assess its efficacy and limitations in these patients.

The results of this study showed that improvement in the experimental group was statistically significant in comparison with the control group. The results on day 3 and day 7 (Tables 1–3) also show that the improvement was statistically significant. In addition, there were no clinically significant abnormalities in either the biochemical or haematological parameters which would have required discontinuation of the study.^12^,^15^ Moreover, the response on CGI-S was also significant in the experimental group compared with the control group (Table 4). Hence, injectable valproate is more efficacious and better tolerated than oral valproate as it reduces manic symptoms more rapidly than oral valproate (Table 3), i.e. on day 3 and day 7. The overall hospital stay was also reduced in the experimental group. The authors recommend that injectable sodium valproate may be used as a first-line drug in the management of acute manic excitement.

**Table 4:** Improvement of clinical global impression scale

	Experimental	Control
Very much improved	8	4
Much improved	10	10
Minimal improved	6	8
Becomes worse	2	4
No change	4	4
